# Automated Item Generation: impact of item variants on performance and standard setting

**DOI:** 10.1186/s12909-023-04457-0

**Published:** 2023-09-11

**Authors:** R. Westacott, K. Badger, D. Kluth, M. Gurnell, M. W. R. Reed, A. H. Sam

**Affiliations:** 1 Birmingham Medical School, University of Birmingham, Birmingham, UK; 2 Imperial College School of Medicine, Imperial College London, London, UK; 3 Edinburgh Medical School, The University of Edinburgh, Edinburgh, UK; 4 Wellcome–MRC Institute of Metabolic Science, University of Cambridge and NIHR Cambridge Biomedical Research Centre, Cambridge University Hospitals, Cambridge, UK; 5 Brighton and Sussex Medical School, University of Sussex, Brighton, UK

**Keywords:** Assessment, Automated item generation, Multiple choice questions, Standard setting

## Abstract

**Background:**

Automated Item Generation (AIG) uses computer software to create multiple items from a single question model. There is currently a lack of data looking at whether item variants to a single question result in differences in student performance or human-derived standard setting. The purpose of this study was to use 50 Multiple Choice Questions (MCQs) as models to create four distinct tests which would be standard set and given to final year UK medical students, and then to compare the performance and standard setting data for each.

**Methods:**

Pre-existing questions from the UK Medical Schools Council (MSC) Assessment Alliance item bank, created using traditional item writing techniques, were used to generate four ‘isomorphic’ 50-item MCQ tests using AIG software. Isomorphic questions use the same question template with minor alterations to test the same learning outcome. All UK medical schools were invited to deliver one of the four papers as an online formative assessment for their final year students. Each test was standard set using a modified Angoff method. Thematic analysis was conducted for item variants with high and low levels of variance in facility (for student performance) and average scores (for standard setting).

**Results:**

Two thousand two hundred eighteen students from 12 UK medical schools participated, with each school using one of the four papers. The average facility of the four papers ranged from 0.55–0.61, and the cut score ranged from 0.58–0.61. Twenty item models had a facility difference > 0.15 and 10 item models had a difference in standard setting of > 0.1. Variation in parameters that could alter clinical reasoning strategies had the greatest impact on item facility.

**Conclusions:**

Item facility varied to a greater extent than the standard set. This difference may relate to variants causing greater disruption of clinical reasoning strategies in novice learners compared to experts, but is confounded by the possibility that the performance differences may be explained at school level and therefore warrants further study.

## Background

Multiple choice question (MCQ) examinations are a mainstay of knowledge assessments within medical education and when constructed well, have been shown to be a valid and reliable testing tool that can appropriately measure clinical reasoning skills and higher order thinking [1–5]. They enable a large amount of content to be tested in a short amount of time and are cost efficient to administer as they can be computer-delivered and machine-marked [6]. MCQs have also been shown to correlate well with other measures of cognitive ability [7] and to discriminate between high and poorly performing students [8, 9]. Trends in medical education, such as computer adaptive testing, programmatic assessment, and progress testing have resulted in the need for an ever increasing number of high quality MCQ items [10]. Furthermore the move toward online assessment delivery brings additional challenges in maintaining the security of the item bank [11, 12]. Developing high quality test items using the traditional method of curating experts to author, review and quality assure items is both time-consuming and expensive. Rudner [13] estimated that a single item cost $1500—$2500 to develop. It is perhaps not surprising therefore, that Automated Item Generation (AIG) is gaining interest within healthcare education as a way of efficiently increasing the size of a question bank, thereby limiting individual item exposure and ameliorating the effects of question leakage.

AIG uses computer software to derive multiple test items from a single question model (the parent item). Lai et al. [14] describe the process of AIG as requiring an expert to develop the original question model or template and then defining the characteristics of the question which can be manipulated to create new items. The computer then generates new items based on the characteristics identified for manipulation. The most straightforward model creates clones of the original question by identifying parameters within the item that may be altered to generate variants. For example, where the item stem refers to pain in the knee, content experts may input additional joints (e.g. shoulder, hip) as variants. This method relies on substitution of information within the vignette and the variation on psychometric properties is expected to be small as the same learning outcome is being assessed within the same context, thus creating ‘isomorphic’ variants. The number of new items that can be generated from a parent question depends on the number of characteristics and range of variables which can be manipulated from any given parent item. The items created during this process have the same ‘template’ of information as the parent item, for example each would have the type and quality of the pain, the past medical history, the temperature, pulse and blood pressure etc.

AIG items have been shown to create items of similar quality as questions traditionally crafted by content experts [15–17]. Gierl et al. [18] have also shown that AIG items possess similar psychometric properties to MCQs constructed using the traditional single item development and review process. Shappell et al. [19] (2021) specifically looked at the test – re-test effect of using AIG to create two 20-question ‘isomorphic’ test papers that were sequentially sat by 47 emergency medicine residents and found a high level of consistency for pass / fail decisions.

When reviewing a parent item for use in AIG, the variables for manipulation can be defined as ‘radical’ whereby changing the variable alters one or more of the content, context or difficulty of the item or ‘incidental’ whereby changing the variable creates a variant with the same presumed content and difficulty [20]. Questions that differ only in incidentals are often called ‘isomorphs’ or clones [21]. Drasgow et al. [22] describe the creation of AIG items in terms of weak and strong theories. In weak theory, an existing question with good psychometric properties is chosen as the parent item. This item then has surface features manipulated which are not expected to change how the student processes the item characteristics. In strong theory, AIG aims to ‘generate calibrated items automatically from design principles by using a theory of difficulty based on a cognitive model’ [22], however this theory requires a knowledge of the variables that impact item difficulty.

In the UK, the Medical Schools Council Assessment Alliance (MSCAA) works collaboratively across medical schools to maintain a bank of high quality assessment items and an electronic exam delivery platform through collaboration with a software development company (epiGenesys®). The items in the question bank are designed to test clinical reasoning or application of knowledge, and many questions have psychometric data from previous assessments. We developed software to automatically generate new variants from existing items using ‘weak theory’ methodology, similar to that of the item clone method described by Lai et al. [14]. Our software enabled implementation of multiple incidental variations from a parent item and so enabled high a high number of clones to be created, which were presumed to be isomorphic.

There is currently a paucity of literature looking at AIG variants at item level in medical education to identify which parameter alterations yield differences in both student performance and standard setting behaviour. The purpose of this study was therefore to use questions from the MSCAA question bank to generate four isomorphic 50-question MCQ assessments using AIG for use as online formative assessments by UK medical schools to:

i) Compare performance data across four test papers
ii) Compare standard setting data across four test papers
iii) Compare the standard setting data with the performance data
iv) Analyse question variants that have a significant discrepancy between student performance, standard setting or the difference between student performance and standard setting

We hypothesized that manipulation of some variables would have a greater effect on standard setting and student performance than others. The aim of the question analysis element of the study was to identify themes that may affect item performance, which could help refine AIG template models and aid better prediction of item performance in future.

## Methods

### Software development

The software company, epiGenesys®, worked with KB and RW to create and embed AIG within the functionality of the pre-existing MSCAA assessment platform (ExamWrite®). An iterative software development process was used to enable the implementation of numeric (e.g. age range) and descriptive (e.g. description of symptoms) parameters. The software then generated variants via computer algorithms selecting combinations of variables within the parameters set for each question. The software was programmed to enable the linkage of gender-based phrases i.e. once gender was selected, it would change all the pronouns within an item to match the stated gender.

### Item and assessment creation

**Fig. 1:**
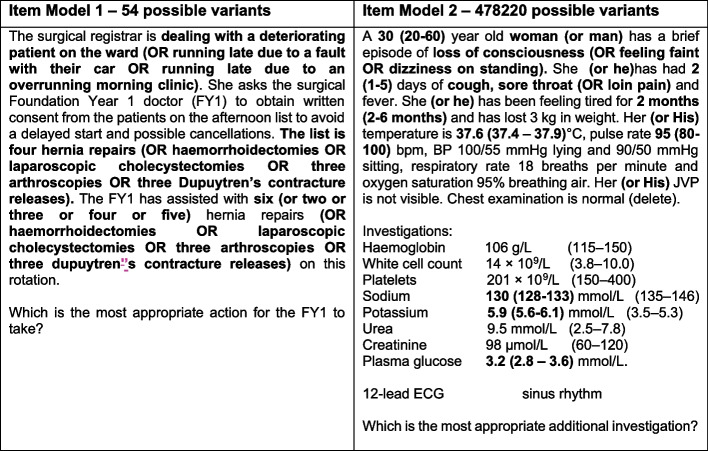
Example item models and possible number of item variants that could be produced from parameters set within the item. Descriptive and numeric variables used to create variants in the item are highlighted in bold font, with parameters and variables shown in brackets (). Note: Haemoglobin is also corrected for male and female but not shown here

Fifty items were identified within the MSCAA item bank for use as item models. Items were all suitable for a final undergraduate medicine examination. They were selected on the basis of having sufficient clinical information to allow the creation of distinct variants and covering as broad a blueprint as possible. The original items were written using traditional item writing methods and followed a single best answer format, with a stem, lead-in and five answer options. Items were written to maximise clinical reasoning and application of knowledge while minimising the cognitive load for students. As a result, the structure and length of the individual item stems varied considerably, leading to significant differences in the type and amount of clinical detail contained and therefore the number and type of variables that could be manipulated to create question variants (Fig. 1). Items with longer stems and items containing clinical observations or investigation results tended to generate the most variants.

Parameters for variables were initially set by KB and RW, and subsequently agreed with all authors using consensus methodology. The AIG software was set to generate and list fifteen item variants for each item model. These were manually reviewed by KB and RW and four variants displaying maximum difference from each other were selected for inclusion into the assessment papers. Four distinct 50-item question papers (A-D) were subsequently compiled, with care taken to ensure a range of age and gender representation in each paper (A-D).

### Delivery of assessment

All UK medical schools with students sitting final undergraduate medicine examinations were invited to participate. Students were required to sit the assessment online using the MSCAA exam delivery platform. Participating schools were randomly allocated one of the four papers (A-D) which was administered online during a one-hour timeslot without breaks, unless students had an agreed reasonable adjustment which was then accommodated. The assessment could be sat remotely but was required to be delivered under the exam conditions set out by each individual medical school. All student performance data was collected automatically and anonymously via the MSCAA assessment platform.

### Standard setting

Each paper was standard set on the MSCAA assessment platform using a modified Angoff method [23] by a separate nine-person panel, eight of whom were members of the MSC national standard setting panel and one of whom was a clinical teaching fellow. The Angoff method of standard setting requires each rater to estimate the likelihood that a minimally competent student would get the question correct. The scores are averaged across the raters for each question and then all average item scores summed to determine the pass mark for the paper. A modified Angoff method is used to describe any modification to this model and many different modifications exist [24, 25]. In this study, the Angoff method was modified to include question answers and to show performance data where this existed. Standard setters only saw one item variant produced from each item model. All standard setting groups also scored a set of 30 common items in addition to the 50 item variants to enable comparison of standard setting behaviour between the four groups.

### Analysis of performance data

Item response data was automatically available from the MSCAA assessment platform. Psychometric analyses were carried out for each individual paper using classical test theory as this was already inbuilt into the ExamWrite® platform. Each test was also compared with the other three assessments, including the mean facility (difficulty), Cronbach’s alpha, and Standard Error of measurement.

Items with low item facility on performance data (questions where a minority of students got the question correct) or which had a negative point bi-serial (less able students were more likely to get the question correct) were reviewed. If a problem was found with the question, it was removed before subsequent analysis of the data at individual item variant level.

At individual item variant level, facility was compared for each item against the other three variants of the item model and these were also compared to the standard set (Angoff score) by the expert panel. Item models containing variants with a difference in facility of > 0.15, or any difference in Angoff score of > 0.1 were identified and the variation in their parameters were explored qualitatively. A greater threshold for analysis was set for facility than for the standard set as there was a greater variation within this parameter.

Items with a facility difference of > 0.15 underwent qualitative analysis using comparison tables. Each variable was scrutinised across each of the four question variants to explore which parameters were most likely to have led to a difference in performance. This was a qualitative hypothesis-generating process considering how variables may impact clinical reasoning, and test-taking behaviours. KB and RW independently reviewed the data and subsequently used consensus agreement to generate initial hypotheses for those parameters that were most likely to have resulted in a significant difference in performance.

## Results

The four papers were sat by a total of 2218 students from 12 medical schools (Table 1).

**Table 1:** Number of students and medical schools sitting each AIG exam

AIG exam	Number of students	Medical Schools Code
1	444	B,C,D
2	541	E,I
3	574	F,G,K,L
4	659	A,H,J
Total	2218	12

### Item review process and removal of items from the exam

Items with poor item facility on performance data or a negative point bi-serial were reviewed before any other analysis of the data was undertaken. This resulted in the removal of three questions from the papers before reviewing individual questions. Performance data highlighted that two of the items had two correct answers (Q15 and Q22). An additional question (Q10) with very poor performance was felt to both be postgraduate rather than undergraduate knowledge and to have a lead-in that lacked clarity in the calculation it was asking for. A further question was removed during qualitative analysis of the questions but did not flag with performance data. This fourth question (Q11) was removed as the AIG process created variants for this particular item that enabled there to be more than one correct answer, however this was only revealed on reviewing each of the variants. Standard setting data and average student performance were subsequently re-calculated based on the 46 items that remained in the assessments.

### Analysis of exam performance

The four tests had acceptable internal consistency with Cronbach’s alpha values 0.67 – 0.75: Paper 1, 0.75 (SEM 3.01), Paper 2, 0.72 (SEM 3.05), Paper 3, 0.67 (SEM 2.93) and Paper 4 0.71 (SEM 3.11).

There was variation in the average facility of papers (range of 0.55 – 0.65), with comparatively smaller variation in the average standard setting results (range 0.58 – 0.61) (Table 2). Of note the standard set for each paper is close to the average student score and actually higher than the average student score for paper 4.

**Table 2:** Average paper facility and standard set using a modified Angoff method (46 questions)

AIG exam Paper	Average Facility	Standard set
1	0.61	0.58
2	0.59	0.59
3	0.65	0.61
4	0.55	0.58

The four different standard setting groups showed good consistency when marking the same 30 items (Table 3).

**Table 3:** The standard set for the common 30 items distributed to the four separate standard setting panels

Standard set	Group 1	Group 2	Group 3	Group 4
30 Common Items Paper	0.60	0.60	0.59	0.60

Following removal of four questions, student performance data was compared to standard setting data for each of the four papers, the results of which are shown in Table 4. Those highlighted in pink had a facility range of equal or greater than 0.15 between the item variants. Those highlighted in lilac had a difference in standard set of equal or greater than 0.1 between item variants. Some questions had variance highlighted in both student performance and standard set.

**Table 4:**
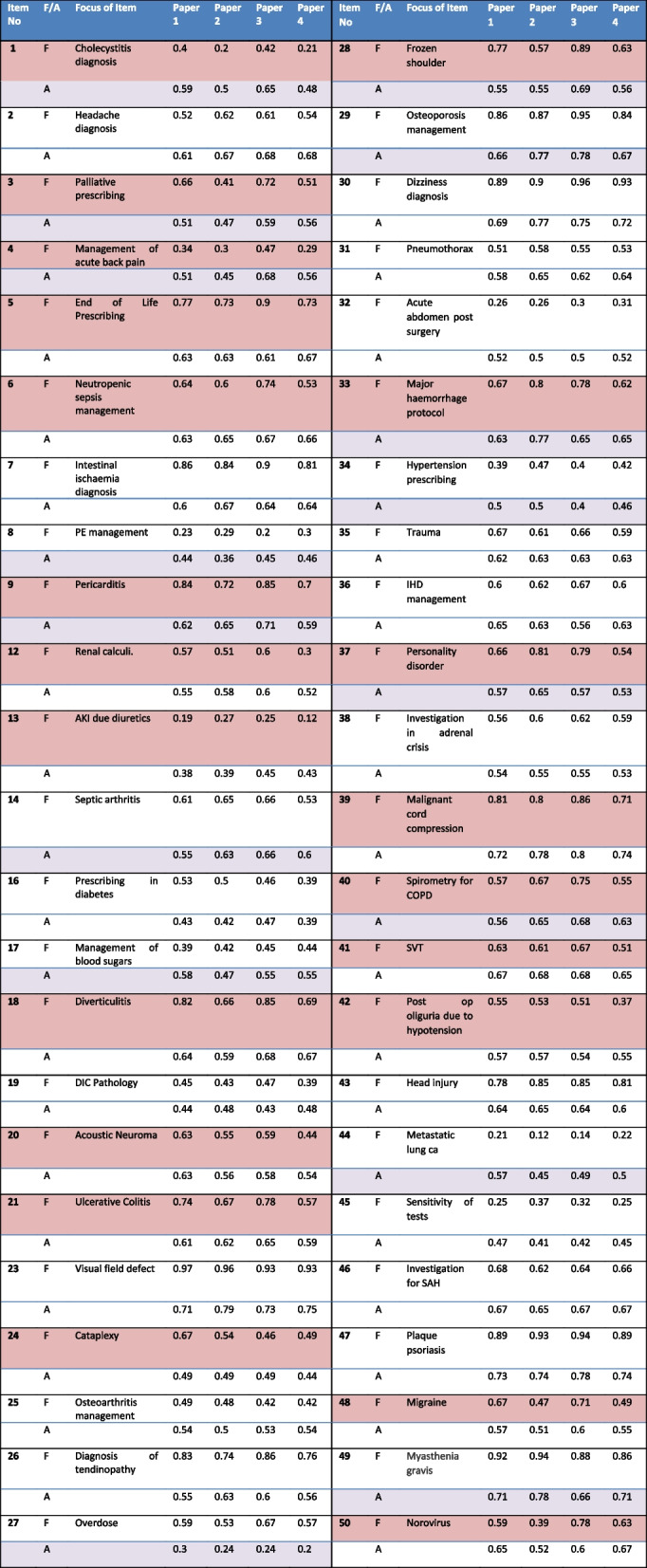
Facility (F) and Angoff (A) scores for Item Models. Pink = Facility range ≥ 0.15 between item variants. Lilac = Angoff range ≥0.1 between item variants

There was a good correlation between item facility and Angoff scores for each of the 4 papers (Paper 1: *r* = 0.65, Paper 2: *r* = 0.83, Paper 3: *r* = 0.68 and Paper 4: *r* = 0.67).

Qualitative analysis of item variants was carried out for the 21/46 item models with a facility difference ≥ 0.15 and the 16/46 item models with a difference in standard setting of ≥ 0.1. Out of the 21 items with facility difference ≥ 0.15, eight also showed a difference in standard setting of  ≥ 0.1. Sixteen items had a difference in Angoff scores of  ≥ 0.1 across the four variants. Angoff scores demonstrated lower levels of variance than facility: the average range of variation across the four variants was 0.03 for Angoff scores compared to 0.10 for the variance in facility, although the variation differed markedly between questions (as shown in Table 4).

**Fig. 2:**
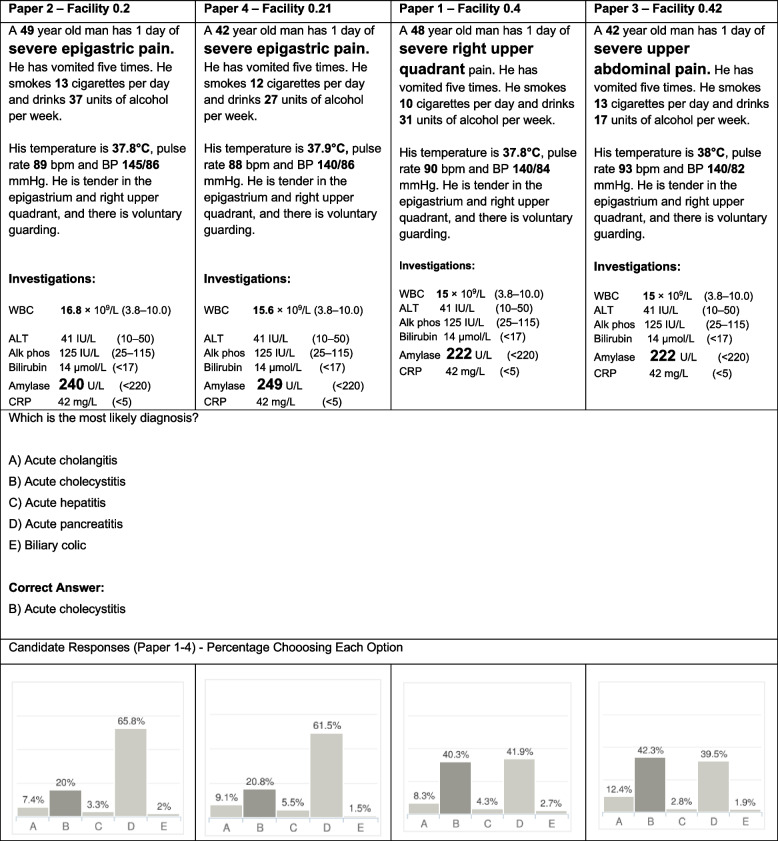
Item number 1 variants across four papers. Text in bold are parameters altered between item variants and enlarged font text indicates the parameters proposed to be causing most variation in facility. WBC: white blood count; Alk phos; alkaline phosphatase

**Fig. 3:**
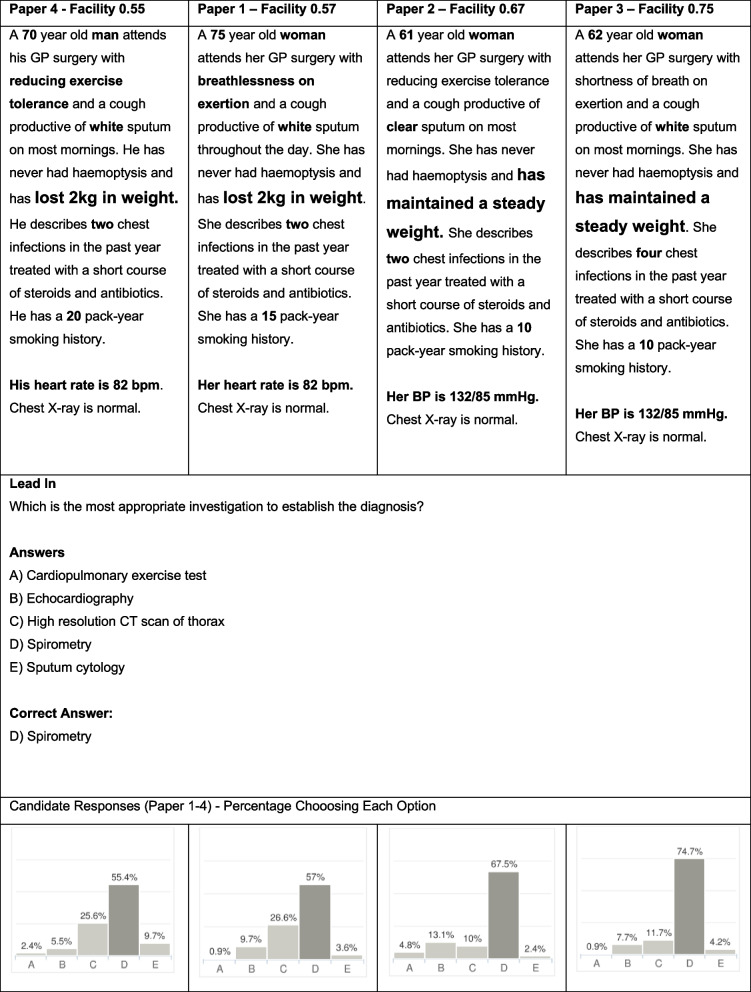
Item number 40 in the exam paper with the four variants shown. Text in bold are parameters altered between item variants and enlarged font text indicates the parameter proposed to be causing most variation in facility

A potential reason for a difference in performance was identified in 14/21 of the items with no clear cause being found in the remaining 7 questions. Factors hypothesised to affect facility tended to be those that created difference in the typical description of a condition. Item 1 demonstrates the impact of changing parameters in the vignette in which the patient has acute cholecystitis (Fig. 2). The two variants which describe the location of pain as the “right upper quadrant”, the stereotypical location of the pain in acute cholecystitis, also by chance had lower amylase levels and both factors are likely to have contributed to the higher facility. The items with a description of severe epigastric pain which also by chance had marginally higher amylase levels more frequently led students to think the diagnosis was pancreatitis (option D). In a second example (Fig. 3, item 40 in the paper) asking for the most appropriate test to establish a diagnosis of COPD, a small but insignificant amount of weight loss appears to have been the variable most associated with item facility. The two item variants where the patient has “lost 2 kg in weight” have a lower facility than variants where the patient has “maintained a steady weight” and resulted in a higher proportion of students choosing to order a CT scan which would be most appropriate investigation if a diagnosis of cancer is suspected.

**Fig. 4:**
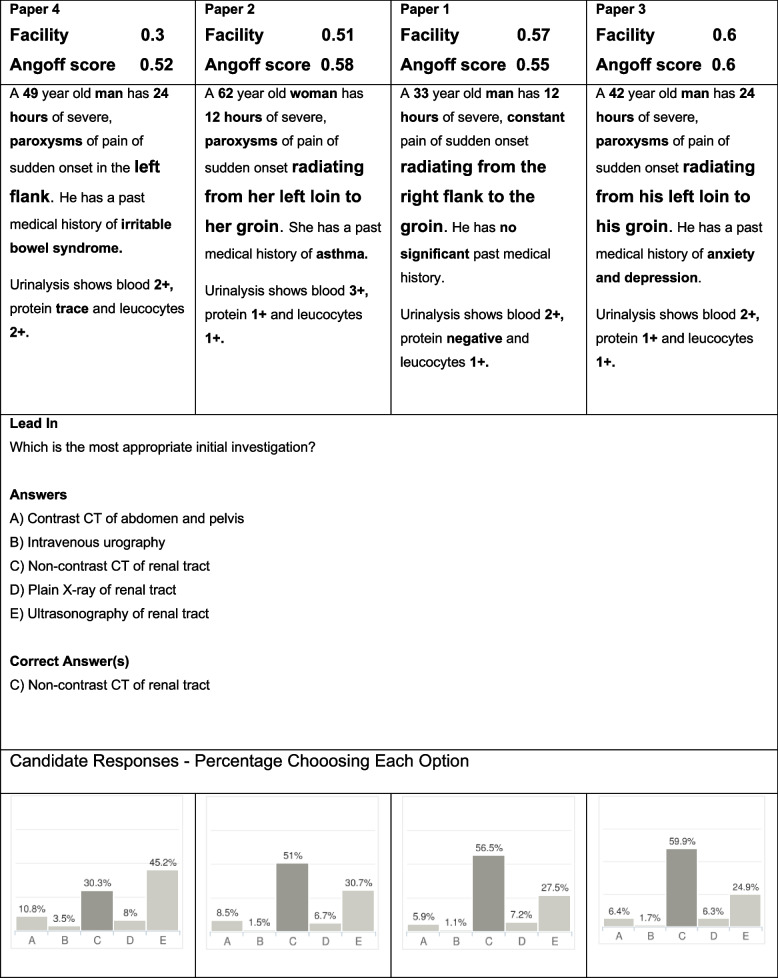
Item 12 variants across 4 papers with facility and Angoff score listed. Text in bold are parameters altered between item variants and enlarged font text indicates the parameter proposed to be causing most variation in facility and standard setting

The trend of lower variance in standard setting data in relation to facility is demonstrated in Fig. 4 which displays an item model asking the candidate to select the best investigation for a patient with kidney stones. The variants demonstrate a similar trend in standard setting and facility scores. The standard setting range of 0.08 (0.52–0.60) is much lower than the facility range of 0.30 (0.30–0.60). Qualitative analysis of both the facility and standard setting score of the item appears related to the presence of the prototypical description of renal colic pain location; “loin (or flank) to groin”.

## Discussion

This study used a new, bespoke programme within ExamWrite® (the question storage and delivery platform created by epiGenesys for the Medical Schools Council Assessment Alliance) to generate four separate 50-question MCQ assessments based on weak theory or an item clone model of AIG, which was similar to that described by Lai et al*.* [14]. Each question within the MSCAA question bank contains the minimum amount of information required to derive the correct answer so that cognitive load is reduced as much as possible rather than having a generic question template for each clinical vignette. This inevitably creates variability in the amount of content and overall length of each question. It is perhaps not surprising that items containing more information, and in particular those containing both examination findings and investigation data, had the potential to create the greatest number of variants.

The assumption was that by altering incidental variables, ‘isomorphic’ items should be produced. The study therefore aimed to test whether the four AIG papers would have the same facility and standard set. The results however, suggest that ‘isomorphic’ MCQ assessments created using AIG do not necessarily have the same facility when given to students of similar ability as demonstrated by the fact that the average student performance varied by 10% across the four papers, from 55% (paper 4) to 65% (paper 3). This contrasts with a much smaller difference in the standard set for each paper, where the passing standard set only varied from 58% (papers 1 and 4) to 61% (paper 3).

As the cohort sitting each assessment was relatively large (444 to 659 students), it seemed reasonable to assume that the spread of student ability across the four papers would be similar. The assigned passing standard for each paper is consistent with this notion, with only small variances between papers. Importantly however, the average facility returned for each paper showed significant divergence. There are a number of alternative explanations for the differences observed in overall performance. Firstly, assessment papers were assigned per medical school and not randomly allocated to individual students. There is evidence showing that performance in MCQ assessments varies between institutions within the UK [26] and this may be one explanation for the difference in performance. The AIG papers were also sat within a relatively narrow time window of six weeks, resulting in student cohorts from different medical schools having a variable amount of time between sitting this formative assessment and their subsequent summative examinations. Different medical school cohorts are therefore likely to have been at different stages of exam preparation when they took this assessment. Students may also have varied in terms of their individual level of engagement with this online assessment depending on their approach to formative assessment opportunities. Unlike the standard setting groups who had a set of common items to use as calibration, the AIG papers sat by the students did not contain any common items, thus no comparison of ability between the groups was possible.

As mentioned above, the standard set for each paper showed far less variation than student performance but approximated the average student performance for each of the four papers. It is well known that those setting the passing standard using the Angoff method have a tendency to revert to the mean [27]. The findings of a previous large study looking at standard setting in Australian medical schools [28] also showed the same trend for standard setters to underestimate the difficulty level of hard items and overestimate the difficulty level of easy items, with implications for how well standards then correlate with actual student performance. Our study also showed significant reversion to the mean, in that on average judges underestimated the facility of easy questions and overestimated the facility of difficult questions. The standard set using the Angoff method should be that of a borderline candidate (a minimum passing score), which with a normal distribution of ability would mean that the majority of students should pass. If the standards set for our study were applied, around 50% of the total cohort would fail. The initial assumption could be, that the standard is too high and that those standard setting did not apply an appropriate standard for a final medical undergraduate examination. However, the members of the standard setting panel were drawn from a national standard setting group that have set a reliable standard for common content items used for final year examinations in medical schools across the UK in previous years. The more likely explanation is that success in MCQ examinations is significantly dependent on student preparation and participants in this formative study had not completed their pre-examination studies and are therefore likely to have had a lower level of knowledge than they would when they sit their summative assessments.

It was not just in the overall scores that student performance differed. Individual questions showed significant variation both in terms of how each of the four variants performed relative to each other and also, in the degree of correlation between student performance and the passing standard set (highlighted in Table 4). In seeking hypotheses to explain this finding, the predominant theme identified was that the facility of question variants diverged most when clinical vignettes deviated more from typical keywords (or ‘buzzwords’) associated with the condition. The facility was lower for vignettes with greater deviation from the prototypical description of signs and symptoms than for the question variants that had a more classic description. Importantly, those standard setting did not appear to anticipate the degree of difficulty that this type of variant would engender. There are several possible explanations for this observation. The illness script is a concept that was introduced by Feltovich and Barrows [29] to explain how doctors make diagnoses. An illness script consists of the typical components and general sequence of events that occur in a specific disease and once established, illness scripts allow automatic activation of pattern recognition [30]. Script ‘instantiation’ occurs each time a physician sees a patient with a given condition, therefore each patient seen helps to refine the general illness script for that condition, for each individual clinician. Clinicians with more experience develop more refined illness scripts in particular with regard to associated ‘enabling conditions’ (the patient and contextual features such as age, risk factors etc. that influence the probability of that condition being the diagnosis). It is likely that those setting the standard for the questions have more developed illness scripts and more readily arrive at the correct answer regardless of the text variants used, and therefore give each variant a similar standard. On the other hand, students will have less well developed illness scripts, have a more rudimentary organisation of events, and may rely more on prototypical descriptions of individual signs and symptoms when reaching an answer [30, 31]. Therefore when a student’s knowledge is based mainly on learning prototypical descriptions (using keywords or buzzwords) rather than clinical experience, their pattern recognition of a given condition is likely to be less developed so they lack awareness of the variability in disease presentation seen in the real world [32]. This is exemplified by Item 12 (Fig. 3), where student performance is hypothesised to be related to the use of the phrase ‘loin to groin’ which acts as a buzzword for renal colic. In support of this hypothesis, Chan and Eppich [33] found that doctors equated keywords (or buzzwords) with studying for undergraduate medical examinations and one of the participants in their study, a junior doctor said when we’re first learning clinical medicine, a lot of the patterns that we recognise are in specific phrases’ [33]. They concluded that keywords can communicate entire diagnoses and activate illness scripts independently of any other information. Think aloud studies looking at approaches to answering multiple choice questions have also identified recognition of buzzwords as a test-taking cognitive approach to answering questions [34]. Sam et al. [35] identified the response to buzzwords as a test-taking behaviour leading to superficial non-analytical cognitive processes in their think aloud study looking at the cognitive approaches students use to answer written assessments.

An alternative way to interpret the observed finding of a lower facility in variants containing less prototypical descriptions of a condition, is to consider cognition errors in the context of dual processing and bias. Norman [36] describes ‘representativeness’ as a form of bias, which is the tendency to be influenced by the prototypical features of a disease and risk missing an atypical presentation. An example of this is shown in Fig. 2, where students were more likely to correctly diagnose acute cholecystitis if there was a prototypical description of right upper quadrant or upper abdominal pain but were less likely to make the diagnosis if the pain was described as epigastric, even when other evidence supported this diagnosis. Representativeness bias was also demonstrated in a study by Rathore et al. [37] in which two role players (one a white man and the other a black woman) both presented with identical symptoms of ischaemic heart disease and students were less likely to characterise the black woman’s symptoms as angina than the white man’s (46% vs 74% for the white male patient, *P* = 0.001). Croskerry [38] describes a number of different cognitive errors including premature closure, which is the acceptance of a diagnosis based on initial salient information and without consideration of the whole presentation. Overreliance on keywords can result in premature closure, if the keyword(s) is/are assumed to verify the diagnosis [33] and premature closure due to honing in on keywords has also been cited as a cause of cognitive error when answering MCQs [39, 40]. In this study we also found that changing a keyword or phrase could potentially invoke a false illness script as shown in Fig. 3, where the undue emphasis on weight loss (even though it is only a modest amount) was thought to have made a significant number of students erroneously consider cancer as the most likely diagnosis, as this would be the primary reason for requesting a CT scan.

Whilst this study has demonstrated that question variants created using AIG (and presumed to be isomorphic) have different psychometric properties, we acknowledge that there were limitations to the study. Firstly, participants were randomised by medical school and therefore the time between sitting this assessment and the final summative examinations were different between different medical schools and this may have impacted on student performance in this assessment. Furthermore, we know that performance in common content items in summative examinations also varies between medical schools across the UK [26]. Therefore, differences in performance between papers may be a result of difference in school cohort performance rather than question characteristics. The common content items used for standard setting were part of a secure question bank that were not available for formative assessment due to concerns regarding item security however using common content items in the student assessment papers would have helped identify whether the differences between papers were a function of the question items or overall student ability. Secondly, whilst the study set out to investigate whether differences in performance and standard setting were observed, it was not designed to test any specific hypotheses as to why this might occur.

## Conclusions

This study has shown that the AIG functionality used in this study represents a potential way to increase the size of a national question bank for summative assessments. We believe this study is the first to demonstrate that item variants produced by changing incidental variables (creating clones) using AIG leads to wider variation in student performance than in standard setting behaviour. This study demonstrates that ‘isomorphic’ (or clone) questions generated by AIG for undergraduate medical assessments should not be assumed to have the same passing standard, and therefore each variant should be standard set as an individual item.

We also offer a possible explanation for this phenomenon in terms of illness scripts, reliance on keywords and the resultant bias that can be created. Further research into the effect that using or avoiding keywords and prototypical descriptors has on student performance and standard setting behaviour is warranted. Anchor items should be used in future studies if using different student cohorts to allow test equating and to increase the confidence that observed differences are the result of question characteristics rather than a difference in cohort ability.

## Data Availability

The datasets used and/or analysed during the current study are not publicly available as they contain potentially sensitive data but are available from the corresponding author on reasonable request.

## Abbreviation

AIG: Automated Item Generation
